# Confluent Extended Posterior Left Atrial Wall Ablation: Thinking Inside the Box

**DOI:** 10.19102/icrm.2017.080704

**Published:** 2017-07-15

**Authors:** M. Clive Robinson, Lindsey Scierka, Murali Chiravuri, Robert Winslow, Rafael Squitieri, Albert Dimeo, Richard Feinn, Joseph J. Tiano, Craig Mcpherson

**Affiliations:** ^1^Department of Cardiothoracic Surgery and Department of Cardiology, Bridgeport Hospital, Bridgeport, CT; ^2^Department of Cardiothoracic Surgery and Department of Cardiology, St. Vincent’s Medical Center, Bridgeport, CT; ^3^Frank H. Netter MD School of Medicine at Quinnipiac University, North Haven, CT; ^4^Yale New Haven Hospital, New Haven, CT

**Keywords:** Atrial fibrillation, Convergent procedure, endocardial ablation, minimally invasive surgery, surgical ablation

## Abstract

Here, we report intermediate follow-up details after using a technique of confluent posterior left atrial wall epicardial ablation designed to eliminate both existing and future atrial fibrillation (AF) substrates. The method is part of the Convergent hybrid procedure for AF ablation. In this study, multiple confluent epicardial ablations with radiofrequency energy were delivered, spanning the vertical and transverse dimensions of the posterior left atrium, along with facilitated pulmonary vein isolation (PVI). Endocardial mapping and ablation were performed to complete PVI and to ablate the cavotricuspid isthmus. All patients were followed clinically and using two-to-four weeks of continuous monitoring at six, 12, and 24 months, respectively. The average length of follow-up was 488 days. Of the 57 largely unselected patients with persistent or longstanding persistent AF (NPAF), mean duration of AF was 5.6 years. Single procedure freedom from AF through 24 months was 64.5%, and that for all arrhythmias, was 58.9%. Sixty-eight percent of patients were off antiarrhythmic drugs. Four patients (7%) required a second endocardial ablation procedure. A sub-analysis of the observed arrhythmia burden present through follow-up showed this to be small (ie, <1%) in the majority of patients involved in this study. In conclusion, the extended posterior left atrial wall ablation technique discussed here, as part of the Convergent hybrid method, achieved notable single-procedure success in a particularly challenging series of patients with NPAF.

## Introduction

The field of interventional therapies for non-paroxysmal atrial fibrillation (NPAF) remains encumbered by many challenges. Despite considerable progress, there is still contention regarding both the quality achieved with individual ablation lesions as well as the type of lesion set required to deal with the complexity of the culprit mechanisms within the arrhythmogenic substrate. Further obscuring the situation is the impact of patient heterogeneity and the many and varied methods by which procedural outcomes are quantified and characterized. These factors confound the ablation experience and efforts to explain and verify both successes and failures, and limit comparisons between clinical series.

As the interventional experience with NPAF has expanded, the Cox maze III procedure, although not curative and often yielding less-than-ideal results in patients with a large left atrium and atrial fibrillation (AF) of extended duration, has maintained credit for the highest and most durable success rate for sinus rhythm.^1^ Based on the definitive “cut and sew” technique and a bi-atrial lesion set, irrespective of its efficacy, and including to a lesser degree the Cox IV and Mini-maze procedures, the current-day perception held by many patients, cardiologists, and surgeons is that the invasiveness of these procedures substantially preclude their general adoptability. As a consequence, the optimal procedure and lesion set has yet to be determined.

Among several components, the primary tenet of the Cox maze III operation is complete posterior left atrial wall and pulmonary vein isolation (PVI). In experiences with other methods to date, achieving these goals has been difficult, and the post-procedure results, disappointing.^2^ With endocardial ablations, there are risks of injury to non-targeted neighboring anatomy and, in traditional endo- and epicardial techniques that create spot and linear lesions, including the box lesion set, there are limitations from partial thickness penetration, gaps, and reconnections.^3^ These shortcomings have led investigators to explore novel multidisciplinary ablation methods that address arrhythmogenic substrates, both existing and future, utilizing endocardial and epicardial approaches together in ways that can be standardized, rendered reproducible, and be well-tolerated by patients.

The purpose of this study was to report on our experience with a technique of extended ablation using a confluent pattern of posterior left atrial epicardial lesions in combination with endocardial ablation, as part of the Convergent hybrid closed chest procedure, performed in a single setting.

## Methods

Fifty-seven consecutive NPAD patients were managed with the Convergent procedure using this confluent ablation technique. All except one had notably advanced NPAF, and were regarded as being either unsuitable for or apt to demonstrate unfavorable results following traditional endocardial catheter ablation. Notably, there was a high proportion of the patient cohort with severe left atrial enlargement, extended duration of AF and obesity. All of the patients had disabling symptoms and had failed to show improvement with conservative measures, including prior antiarrhythmic drug (AAD) use. Data were prospectively accumulated and recorded in a 36-point data registry, which includes our entire experience to date since the introduction of the technique in our institution. This study’s proposal was reviewed by the hospital’s institutional review board and, based on code 214C, we were granted a waiver for obtaining informed consent for patient data acquisition and analysis, although written patient informed consent was still obtained. This study conformed to all relevant parts of the Declaration of Helsinki.

### Preoperative evaluation

Regarding inclusion in this study, patients with structural heart disease at our hospital underwent standard clinical evaluation and were excluded if additional surgical therapies were required. Patients who had undergone any prior cardiac surgery procedures were also excluded. Left atrial and pulmonary vein (PV) morphology and dimensions were assessed using computed tomography (CT) scanning. A transesophageal echocardiogram (TEE) for assessment for left atrial appendage thrombus was performed routinely. Oral anticoagulation use was stopped preoperatively without bridging therapy.

### The Convergent procedure

The Convergent procedure is a hybrid epicardial/endocardial ablation intervention often performed as a single procedure. For the epicardial component, endoscopic surgical methods are used to access the posterior LA surface through a small upper trans-abdominal midline incision with pericardial entry through the central tendon of the diaphragm. A unipolar radiofrequency suction ablation device is used (Numeris^®^ Coagulation System; AtriCure, Cincinnati, OH, USA) to deliver the epicardial lesions. Early versions of this technique utilized limited peripheral box or random ablation sets on the posterior LA wall until the introduction of the extended confluent technique described here, along with facilitated PVI.^4^ For the endocardial component, inaccessible or incompletely ablated epicardial areas are dealt with using standard endocardial mapping and catheter ablation to complete PVI and other non-PV sources if deemed necessary. *Additional details are included in the attached addendum on page 2773*.

### Technique of confluent extended posterior left atrial epicardial ablation

**Figure 1:**
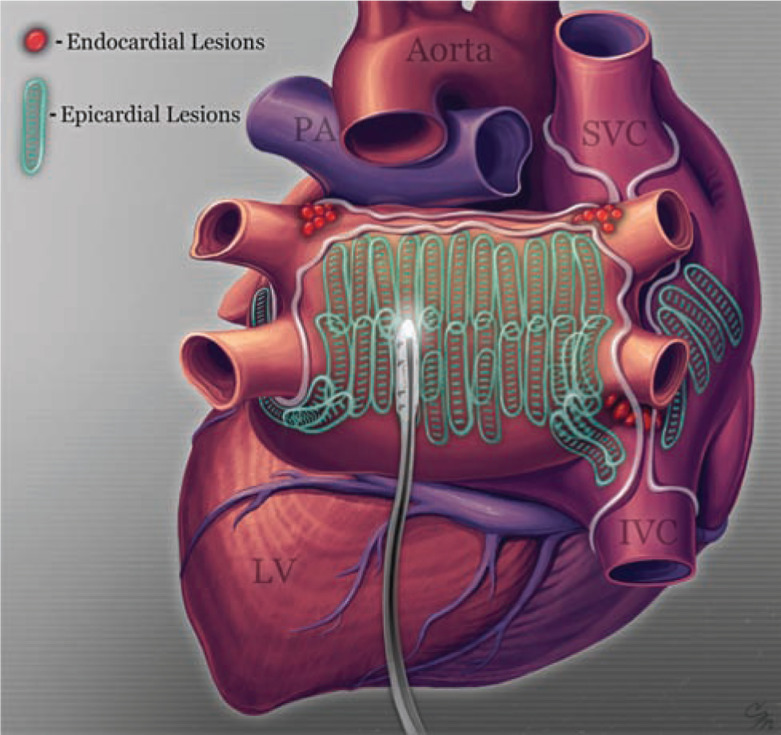
Confluent extended posterior LA lesion set. Courtesy of Cleveland Mosher.

The comprehensive lesion set we developed differs from other epicardial ablations by directly targeting and encompassing all accessible areas of the posterior left atrium within the oblique sinus, as outlined by the pericardial reflections **(Figure 1)**.^5^ The ablations are confluent and span the vertical and transverse dimensions of the posterior left atrium. Superiorly, they are immediately adjacent to the pericardial reflection between the oblique and transverse sinus (roof line); inferiorly close to and following the atrioventricular groove (floor line); and transversely extended across the atrium to be adjacent to the confluences of the PVs. Multiple anteriorly directed and adjoining parallel vertical ablation lesions are placed in this area, and are easily visible on the epicardium. Both the number of epicardial lesions and the number of vertical rows of ablation were determined by the surface area and by particular anatomical characteristics of the posterior left atrium.

Ablation anteriorly was achieved by maneuvering the device from the posterior pericardial space, with separate repositioning on the left and right sides to allow for LA ablation to be completed adjacent to the PV confluence. On the left side, the anterior and posterior ablations were connected around the crux of the inferior PV. Anteriorly on the right, ablation incorporated the fat pad of the inter-atrial groove and its contained autonomic plexi. Continuity of anterior and posterior ablation in the region of the right inferior PV was prevented by the inferior vena cava and adjacent pericardial reflection. Upon completion, the pericardium was drained and the abdominal incision closed.

Following this, the electrophysiology (EP) team then performed the endocardial aspect of the procedure, mapping the posterior wall and completing PVI, as well as ablating other areas as required. A cavotricuspid isthmus ablation was performed routinely. A maintenance dose of heparin was commenced six hours post operation. Oral anticoagulant use was initiated on the morning of postoperative day one. AADs that had been given pre-operatively were typically restarted; otherwise, pharmacological therapy was not prescribed unless necessitated by spontaneous arrhythmias.

### Follow-up protocol

Patients were reviewed by the cardiovascular surgeon and/or the EP cardiologist at weeks one and four postoperatively, respectively, and at intervals of three, six and 12 months following surgery and then every six months thereafter, as well as additionally if needed. These follow-ups included the performance of a 12-lead electrocardiogram (ECG) and use of a continuous electrical monitoring protocol. After the three-month postoperative blanking period, AADs were discontinued unless ongoing atrial arrhythmias were judged to require further therapy. Anticoagulant use was recommended for at least six months post-operation, with patients weaned from such based on their rhythm monitoring results and CHA_2_DS_2_-VASc score.

### Continuous electrical monitoring

Either an ECG event recorder (eCardio Post-Event Recorder; eCardio Diagnostics, LLC, Houston, TX, USA) worn for three to four weeks, or a continuous ambulatory ECG recorder (Zio^®^ Patch; iRhythm Technologies, Inc., CA, USA) worn for two weeks, was used for continuous monitoring at the six- and 12-month intervals following surgery to provide levels of monitoring beyond those used in most clinical series or per Heart Rhythm Society (HRS) guidelines. Compliance rates with post-procedure monitoring were as follows: 53 of 57 patients at six months (93%), 40 of 45 patients at 12 months (89%), and 22 of 25 patients at 24 months (88%).

### Presentation of outcomes

Arrhythmia recurrence was defined per HRS guidelines as the occurrence of post-blanking period AF, atrial flutter (AFL) or atrial tachycardia (AT) lasting at least 30 s, at any subsequent stage of follow-up and documented by any form of monitoring. Arrhythmia burden was defined as any episode of recurrent arrhythmia during continuous monitoring expressed as a percentage of the monitored interval. In those patients where sustained arrhythmias were seen on repeated office visits and ECGs, continuous monitoring was deemed unwarranted, and for the purpose of results analysis, their recurrent arrhythmia burden quantified as being 100%. Patients undergoing a repeat mapping and ablation procedure were identified and accounted in the results as failures. As recurrences can be sporadic through follow-up, and in the absence of continuous implanted monitoring, the presentation of the results includes the following: (1) recurrences collectively carried forward throughout follow-up are shown in the Kaplan Meier curve; and (2) recurrences and key clinical outcomes (including repeat interventions and AAD use) are reported separately as being measured at the prespecified time intervals of six, 12, and 24 months and at the time of the last follow-up of greater than or equal to one year post-operation. For additional insight into outcomes, we also analyzed AF recurrence alone and recurrence of any atrial arrhythmias (specifically, AF, AFL and AT). To assess the durability of the procedure, a sub-analysis focuses on the 44 patients with ≥ one year of follow-up data included in this study.

### Statistical analysis

Descriptive statistics include means and standard deviations for continuous variables and frequencies, and percentages for categorical variables. A Kaplan–Meier curve was constructed to describe AF and AF/AFL/AT recurrence-free survival times. Since patients were followed continuously, including with monitoring performed at discrete time intervals, and could experience an instance of AF or AF/AFL/AT more than once (repeated event),a generalized linear mixed model with a random intercept for patients and a complimentary log-log link function was used to test the statistical significance and estimate the hazards ratio for potential risk factors.

## Results

Baseline information is shown in **Table 1**. With respect to the 57 patients, average follow-up was 488±220 days (range 120 to 843 days). Forty-four patients had an average follow-up of one year or more (average 563 days). Mean duration of AF was 5.6±4.9 years, and 18% of the involved patients were morbidly obese (body mass index (BMI) >40). LA enlargement was prevalent with an average LA volume (identified by CT scan) of 124.4 ml (range 61 to 235 ml), including 40% having ≥130 ml. An average of 27 epicardial ablation lesions were delivered (15–53), with 42 patients receiving two rows and eight patients receiving three rows.

Of the 57 patients in the series, 40 were in AF at the commencement of the procedure. Of these 40, 23 (57,5%) converted to sinus rhythm during the procedure. Ten converted during the epicardial component, with an additional three changing from AF to AFL. Fourteen patients converted during the endocardial part of the procedure, with three of these converting during ablation of the right cavotricuspid line. Of the remaining patients, all were converted to sinus rhythm at the end of the intervention primarily by direct current cardioversion (DCCV), and in two cases pharmacologically using ibutilide.

**Table 2** shows peri-procedural adverse events (which occurred in 7% of patients). There were no recognized cases of stiff LA syndrome. One patient developed congestive heart failure over the first seven to 10 days post-procedure while remaining in sinus rhythm. Despite sinus rhythm, the echo showed a globally paretic left atrium with maintained LV function and no effusion. Ultimately, however, the heart failure resolved and atrial contractility returned within three weeks. No right heart catheterization was performed. Late events (to one year) included three patients with post-pericardotomy syndrome who were managed conservatively, and three with abdominal wall incisional hernias that were repaired. No patient required permanent pacemaker implantation. The average length of hospital stay was 2.9 (2–5) days. The 30-day hospital readmission rate was 3.5%.

**Figure 2:**
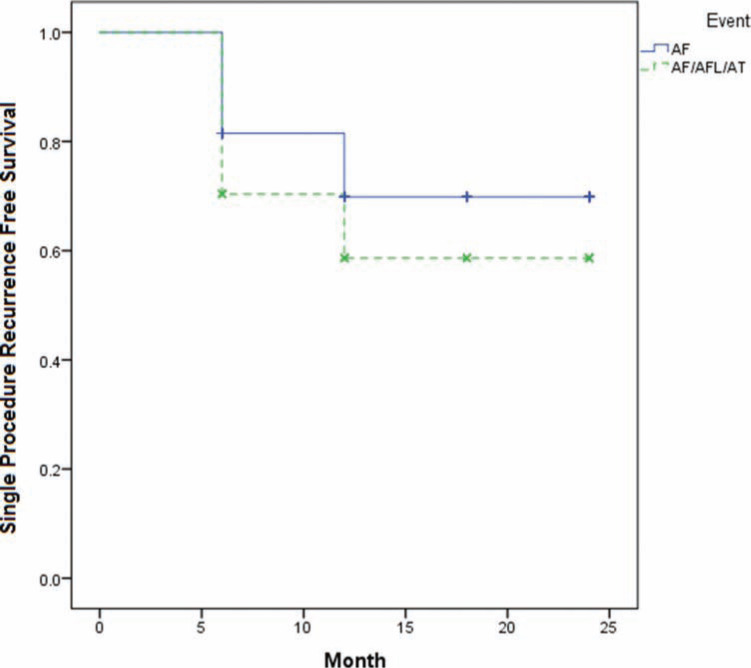
Kaplan-Meier curve of success (according to HRS guidelines) (n=57).

**Figure 2** shows the Kaplan–Meier curves for the 57 patients and records single procedure freedom from (1) AF alone; and (2) from AF, AFL, and AT. For follow-up throughout the two years post-operation, freedom from AF alone was identified as 64.5%, and freedom from all atrial arrhythmias was 58.9%.

**Table 3** documents key clinical outcomes as measured at six, 12, and 24 months, and for those patients with follow-up of one year or more. Follow-up when assessed at 24 months showed a single procedure freedom from AF rate of 82%, and that from AF/AFL/AT of 77%. The rate of freedom from the use of AADs was 68%. For the 44 patients with last follow-up at ≥ one year post-operation, single procedure freedom from AF was 84% and, from AF/AF/AT, was 81%. Freedom in this patient subcohort from the use of AADs was 73%.

**Figure 3:**
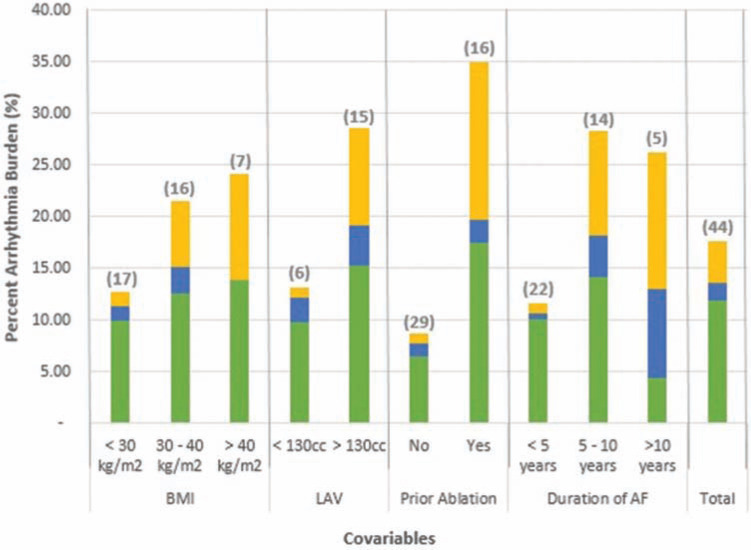
Correlation of covariables with arrhythmia burden in 44 patients with follow-up > one year. The bracketed numbers above the bar represent the number of patients in each category. The height of each bar indicates the average percentage of arrhythmia burden for each covariable category. Within each bar, the color categories represent the proportion of burden for each covariable, and are defined as follows: green <1%, blue 1% to 20%, and yellow >20%.

**Figure 3** evaluates the 44 patients with follow-up of ≥ one year (average: 563 days) to assess the association of covariables (BMI, LA volume, prior ablation, and AF duration) with post-procedure burden of AF/AFL/AT cumulatively throughout the follow-up period. The height of each bar in the figure indicates the average arrhythmia burden in the patient subgroup with the listed covariable. Within each bar, the average arrhythmia burden is sub-divided into categories of <1%, 1% to 20%, and >20% for each covariable. Except for patients in whom AF had been present for >10 years, in all other patient sub-groups, the majority of post-ablation arrhythmia burden was <1%. In general, post-procedure arrhythmia burden was higher in conjunction with an increasing degree of obesity and increased LA volume. Lower arrhythmia burden was attained in patients with no prior ablation attempts, as compared with those who had undergone prior ablation. Arrhythmia burden was significantly lower (p<0.004) in patients with AF duration of <five years. **Table 4** details the statistical significance for each covariable.

Repeat ablation for symptomatic arrhythmias was performed in four (7%) of the 57 patients. All of these patients had AFL and three of them had undergone ablation prior to their Convergent procedure. Restudy with endocardial mapping of these patients revealed the arrhythmia to be located at the upper pericardial reflection of the left superior PV in one patient, the cristae terminalis (with a second focus in the atrial septum) in one patient, adjacent to the atrioventricular node in one patient, and in the mitral isthmus in one patient. Although these patients were all counted as failures, subsequent follow-up monitoring demonstrated a 27% residual arrhythmia burden in one patient and no arrhythmias in the other three.

Notably, no post-procedure arrhythmias were observed to arise from any area in which epicardial ablation had been performed.

**Figure 4:**
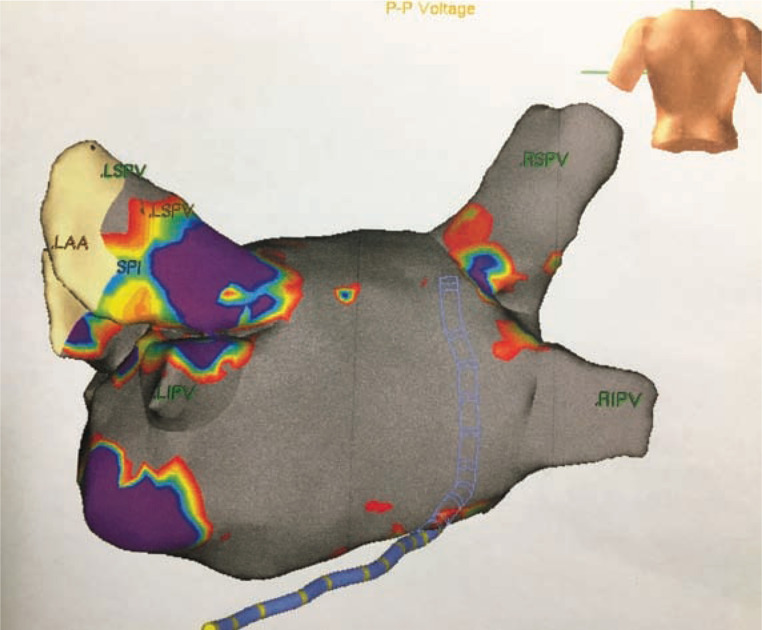
Posterior LA wall endocardial map following confluent epicardial ablation (voltage <0.25 V). The gray portions indicate the electrically silenced atrial substrate.

**Figure 4** shows a typical posterior LA endocardial voltage (0.25 mV) map at completion of the epicardial ablation (Ensite™ NavX™; Abbott Laboratories, Chicago, IL, USA) with substantial silencing of the posterior left atrium.

## Discussion

With NPAF, as structural remodeling advances, the challenges of treatment, including identification, anatomical accessibility and definitive eradication of culprit mechanisms, becomes progressively more difficult, and arrhythmia-free outcomes decline. Beyond the “cut and sew” surgical technique, the conventional use of limited linear and spot ablations in these complex cases, frequently involving suboptimal devices and energy delivery methods, are well-recognized for their deficiencies.^6,7^ The epicardial ablation technique described in this study, in combination with the endocardial component, is designed to circumvent many of these limitations and to target in particular an anatomical area recognized as being a dominant and concentrated site of arrhythmia substrate.

### Procedural technique and rationale

#### Thinking inside the box

Bearing in mind the complexity and limitations of treating advanced NPAF, the epicardial procedure described here was created to achieve a balance between a safe, well-tolerated readily adoptable method that optimizes substrate eradication and minimizes procedural invasiveness, while reducing the need, risk and challenges of extensive endocardial ablation to the posterior wall. Along with this dramatically enhanced posterior wall epicardial ablation, improved safety routines were used, including esophageal temperature monitoring, pericardial irrigation, ablation device stabilization during energy application, and the elimination of approaches involving the pleural cavities and the transverse sinus.

The key features and rationale of this approach as compared with other catheter-based and non-“cut and sew” surgical procedures include the following: that is, the approach provides (1) the ability to more comprehensively electrically silence the posterior left atrial wall than what is achieved with individual linear, spot or box lesions; (2) the ability to assess and uniquely validate the completeness of epicardial lesions via confirmation with endocardial mapping; (3) the ability to deliver extensive confluent anatomical coverage with an ablation pattern that encompasses both existing and future substrates; and (4) the use of a standardized method in which the collective effects of multiple adjoining confluent lesions on substrate may obviate or limit the deficiencies of partial-thickness, arrhythmogenic gaps, PV reconnection and potential proarrhythmic effects seen with individual linear and focal lesions. As a result, conventional dependence on full-thickness penetration with single lesions may be circumvented. Additionally, (5) this approach’s ability to eliminate a large central area of left atrial substrate may offset the need to target adjacent non-ablated sources of arrhythmia (such as the dome of the left atrium in the transverse sinus, the Ligament of Marshall, the lateral ridge, and the LA appendage) that may otherwise require an intact posterior wall to perpetuate; and (6), by reducing the need for extensive endocardial catheter ablation, many of the safety issues of that approach, particularly in large left atria, are overcome or diminished (including char, thrombus formation, silent cerebral emboli, tissue vaporization, phrenic nerve injury, and atrio-esophageal fistula).

The confluent ablation technique used here is standardized and readily adoptable with a straightforward learning curve. Overall, the associated length of hospital stay was short, and the 30-day readmission rate was low. These experiences compare favorably with previously reported Society of Thoracic Surgeons off- and on-pump isolated surgical AF ablation procedures, which have shown complication rates of 13.6% and 27.9% and 30-day re-hospitalization rates of 10.7 and 11.3 days, respectively.^8^

Other than the Cox maze III experience, the alternative methods in current use, including hybrid approaches, continue to be in contention. Reports from these experiences frequently attribute ablation outcomes to lesion set alone, rather than accounting for the importance of lesion quality. As well, amongst published clinical AF studies, there are often widely variable patient characteristics and non-standardized methods of reporting procedural outcomes. Many series fail to clarify whether post-ablation outcomes include just AF alone or other atrial arrhythmias as well. Results are often reported in isolation at the pre-specified time points when measured, rather than recurrences also being reported cumulatively throughout follow-up (as done in Kaplan–Meier curves), and often there is failure to define whether outcomes are from single or repeat procedures. Further, although considered difficult to obtain from a practical standpoint, without pre- and post-procedural mapping information, the explanation of whether recurrences originate from failed initial ablation, from missed sources of arrhythmia arising outside the ablated area, from subsequent new substrate formation, or from pro-arrhythmia effects, often remains unaddressed. As a result of these factors, outcomes between series using the same technique may vary, and the explanation of both successes and failures may be unclear.

Amongst the current-day literature, three notable recent papers relate to these principles. The multicenter STAR AF II trial assessed the success of PVI alone, the combination of PVI and ablation of complex fractionated electrograms, and the combination of PVI and line ablation.^8^ Notably, patients who had undergone prior ablation were excluded. A second study by Gillinov et al. on mitral valve surgical patients with AF compared the success of the performance of PVI alone and the performance of PVI with a bi-atrial lesion set using optional ablation methods.^9^ Surprisingly, both studies failed to show any statistical difference in outcome between the various methods. In contrast, a report by Sirak and Schwartzmann of a large series of surgical endoscopic patients using a standardized bi-atrial lesion set with multiple overlapping reinforcing ablations achieved excellent results.^10^ The inconsistencies seen among these clinical series draws attention to the importance of lesion quality as well as actual lesion set, as the variations in the outcomes between these studies more than likely represent issues with both the quality of the ablations and the actual lesion set used, rather than either on its own. With the lesion set in the Convergent procedure primarily targeting the posterior LA wall and PVI, the question remains as to whether the previously mentioned and well-recognized sources of arrhythmias adjacent to the posterior wall also require direct ablation. Although with this epicardial technique, the superior roofline is delivered as a uniform row of ablation lesions adjacent to the transverse pericardial reflection and the oblique sinus, it does not access or target the true LA roof or dome in the transverse sinus, or the other adjacent arrhythmogenic sources, (including the ligament of Marshall, lateral ridge, and left atrial appendage). The ongoing collection of mapping data and clinical outcomes is needed to clarify this issue.

In the assessment of ablation results, although primary endpoints are traditionally based on absolute arrhythmia recurrence, secondary outcomes that include arrhythmia burden and reintervention rate may better indicate procedural efficacy and the clinical benefit to patients. In this regard, although in the context of our Convergent series being a small and observational study, of those patients who did develop recurrence, the actual arrhythmia burden was, in most cases, dramatically reduced, and re-intervention rate was low.

## Conclusions

When considering the many challenges of ablation in NPAF, the posterior left atrium ranks as a primary and particularly difficult anatomical substrate requiring attention. This paper represents an observational report on our confluent epicardial lesion set used in achieving comprehensive ablation and notable electrical silencing of this area with endocardial mapping confirmation, as part of the Convergent procedure. The study included an advanced group of largely unselected NPAF patients who would usually be rejected for catheter ablation alone and who were assessed by comparatively high levels of clinical and monitoring scrutiny. This paper also underscores the importance of precisely defining patient covariables and methods of outcome monitoring and analysis.

Recognizing the complexity of NPAF and that there is no procedure capable of eradicating all potential AF substrates, the technique described here provides a balance of optimized ablation with minimized invasiveness and risks. The procedure involves an entirely soft tissue approach, and is readily adoptable, well tolerated, and widely applicable. The method achieved notable single procedure success, and adds to the developing ablation experience by providing safe and extensive electrical silencing of this challenging and major area of both existing and future substrates.

## Figures and Tables

**Table 1:** Patient Demographics

Characteristic (n = 57)	Patients (n (%) or Mean ± SD)	Range
Males	44 (77.2%)	
Females	13 (22.8%)	
Age (years)	60.2 ±6.9	46–77
BMI (k/m^3^)	33.0 ±7.9	21.3–55.0
CHA_2_DS_2_-VASc score	1.9 + 1.3	0–5
Paroxysmal AF	1 (1.8%)	
Persistent AF	35 (61.4%)	
LSPAF	21 (36.8%)	
AF duration (years)	5.6 ±4.9	9 months–20 years
Left atrial size (cm)	4.5 ±0.7	3.5–7.1
Left atrial volume (cc)	124.4 ±35.6	61–235
Number of prior cardioversions	4.4 ±3.8	0–25
Number of prior catheter ablations	0.4 ±0.7	0–3

n: number; SD: standard deviation; k/m^3^: kilogram per cubic meter; AF: atrial fibrillation; LSPAF: long-standing persistent atrial fibrillation; cc: cubic centimeter.

**Table 2:** Adverse Events

Peri-procedural Events (< 30 Days) (n = 57) Mortality	Patients (n (%)) 0 (0%)
Myocardial infarction	0 (0%)
Stroke	0 (0%)
Transient ischemic attack	1 (1.8%)
Pulmonary embolus	0 (0%)
Pulmonary vein stenosis	0 (0%)
Atrial esophageal fistula	0 (0%)
Phrenic nerve injury	0 (0%)
Tamponade requiring drainage	2 (3.7%)
Pleural effusion	0 (0%)
Bleeding requiring transfusion	0 (0%)
Femoral artery pseudoaneurysm	1 (1.7%)
Total	4 (7.0%)

n: number.

**Table 3:** Key Clinical Outcomes for Patients at Six-, 12- and 24-Month Time Intervals

	Six Months (n = 53/57)	12 Months (n = 40/45)	24 Months (n = 22/25)	At Last Follow-Up> One Year (n = 44/44)
Single procedure freedom from AFtAADs	84%	83%	82%	84%
Single procedure freedom from AF/AFL/AT± AADs	75%	70%	77%	81%
Single procedure freedom from AF/AFL/AT without AADs	53%	65%	68%	73%
Freedom from AF/AFL/AT±one reablation procedure!AADs	79%	73%	77%	81%
Patients requiring DCCV	12%	10%	6%	6%
Patients requiring re-ablation	4%	5%	0%	0%

n: number; AF: atrial fibrillation; AADs: antiarrhythmic drugs; AFL: atrial flutter; AT: atrial tachycardia; DCCV: direct current cardioversion.

**Table 4:** Generalized Linear Mixed Model Results

Variable	Coefficient	HR	95% CI	p-value
**AF**				
BMI	0.036	1.04	0.97 to 1.10	0.257
AF duration (years)	0.102	1.11	1.02 to 1.20	0.012
Previous ablations	−0.116	0.89	0.42 to 1.90	0.764
LA volume	0.303	1.35	0.68 to 2.69	0.388
**AF or AFL**				
BMI	0.026	1.03	0.97 to 1.08	0.322
AF duration (years)	0.098	1.10	1.03 to 1.18	0.004
Previous ablations	0.170	1.19	0.69 to 2.04	0.538
LA volume	0.338	1.40	0.79 to 2.48	0.244

HR: hazard ratio; CI: confidence interval; BMI: body mass index; AF: atrial fibrillation; LA: left atrial; AFL: atrial flutter.

## Addendum - additional technical details of the Covergent procedure

In all patients in this series, both the epicardial and endocardial ablations were completed in the same procedure, in the order of epicardial and then endocardial ablation.

### Epicardial technique

The procedure was carried out under general anesthesia with standard monitoring. A TEE was performed preoperatively to confirm freedom from LA clots. Defibrillation patches were applied, and an esophageal temperature probe was positioned under fluoroscopic guidance adjacent to the body of the left atrium, 1.5–2 vertebrae below the tracheal carina. Esophageal temperature was continuously monitored during the procedure.

During the epicardial component of the procedure, the pericardial space and left atrium in the oblique sinus was accessed through a 2.5-cm incision in the upper abdominal wall and a small incision in the central tendon of the diaphragm, respectively. For the diaphragmatic opening, standard endoscopic instrumentation was used, including an L-hook cautery probe set at low power. To avoid ventricular irritation, the central tendon of the diaphragm was tented away from the heart during cauterization. Epicardial ablation lines were placed using the Numeris^®^ Coagulation System (AtriCure, Cincinnati, OH, USA). The epicardial ablation electrode of the device is 3×0.6 cm, and maintains direct contact to the target epicardium through suction resulting from negative pressure (−400 mmHg), and includes fluid perfusion for cooling to avoid significant energy delivery beyond the target epicardium. During ablation, room temperature saline was also irrigated into the pericardial space to further ensure only the target epicardium was ablated. Unipolar radiofrequency delivery was maintained for 90 s at 30 watts using an algorithm that detects increases in impedance and reduces power upon increases above a predefined threshold, to prevent tissue vaporization or over-desiccation. In the event of any rapid increase or elevation of esophageal temperature over 1° from baseline, ablation delivery is aborted, pericardial irrigation is increased, and ablation is temporarily moved to another site before subsequent return to that area. In this study, following completion of the epicardial ablation set, a drain was placed in the posterior pericardial space and wound closure was performed. Preparations were then made for the endocardial ablation portion of the procedure.

### Endocardial ablation

At this point, a left atrial electroanatomic navigation map was created using the NavX^®^ system (Abbott Laboratories, Chicago, IL, USA) in combination with the 64-slice, three-dimensional CT scan images obtained during the patient’s pre-procedural workup. Trans-septal catheter placement was accomplished using intracardiac echo guidance. Activated clotting time levels were maintained at greater than 350 s during catheter positioning in the left atrium. A Lasso^®^ catheter (Biosense Webster, Diamond Bar, CA, USA) was used to assess PVI and direct ablation of any residual connections at the pericardial reflections. This catheter was also used to sample the posterior wall following epicardial ablation to interrogate lesion completeness. At the discretion of the EP operator, routine stimulation testing with confirmation of block was performed, along with mapping of the septum, left atrial appendage, lateral ridge, coronary sinus–mitral isthmus; and selected right atrial and superior vena cava mapping. No quantification was made of scar distribution or density.

Endocardial ablation targets included the completion of PVI, any residual active posterior left atrial areas, and the placement of a right atrial cavotricuspid isthmus line. Roof lines, septal lesions, coronary sinus, and mitralisthmus ablation were performed in response to induced arrhythmias as needed, but were not standard. Ablation goals were confirmed using atrial electrograms (AEGs), with success defined as when an AEG diminished by at least 50% during individual ablations. Bidirectional block was confirmed across the PVs and the right cavotricuspid line.

Complex fractionated atrial electrograms were mapped and evaluated based on atrial electrograms, with two or more deflections and fractionated baseline complexes demonstrating continuous activity for greater than 10 s of recording time. Areas of scarring were defined as any region in which bipolar voltage amplitude was less than 0.05 mV.

End outcomes of ablation, either epicardial or endocardial, included either conversion to sinus rhythm or AF converting to an organized AT, including AFL with a similar cycle length in both atria in the coronary sinus, or with AF requiring a direct current electrical shock or conversion pharmacologically. Where AF converted to an organized AT, including AFL, this was mapped and ablated and rechecked 30 min subsequently. In all cases at the end of the procedure, acute procedural success with sinus rhythm was achieved.
